# Effects of Pathogenic Mutants of the Neuroprotective RNase 5-Angiogenin in Amyotrophic Lateral Sclerosis (ALS)

**DOI:** 10.3390/genes15060738

**Published:** 2024-06-04

**Authors:** Giovanni Gotte

**Affiliations:** Biological Chemistry Section, Department of Neuroscience, Biomedicine and Movement Sciences, University of Verona, Strada Le Grazie 8, I-37134 Verona, Italy; giovanni.gotte@univr.it; Tel.: +39-045-8027694

**Keywords:** ALS, angiogenin, ribonucleases, RNase 4, protein aggregation, amyloidosis, neurodegeneration

## Abstract

Amyotrophic Lateral Sclerosis (ALS) is a fatal neurodegenerative disease that affects the motoneurons. More than 40 genes are related with ALS, and amyloidogenic proteins like SOD1 and/or TDP-43 mutants are directly involved in the onset of ALS through the formation of polymorphic fibrillogenic aggregates. However, efficacious therapeutic approaches are still lacking. Notably, heterozygous missense mutations affecting the gene coding for RNase 5, an enzyme also called angiogenin (ANG), were found to favor ALS onset. This is also true for the less-studied but angiogenic RNase 4. This review reports the substrate targets and illustrates the neuroprotective role of native ANG in the neo-vascularization of motoneurons. Then, it discusses the molecular determinants of many pathogenic ANG mutants, which almost always cause loss of function related to ALS, resulting in failures in angiogenesis and motoneuron protection. In addition, *ANG* mutations are sometimes combined with variants of other factors, thereby potentiating ALS effects. However, the activity of the native ANG enzyme should be finely balanced, and not excessive, to avoid possible harmful effects. Considering the interplay of these angiogenic RNases in many cellular processes, this review aims to stimulate further investigations to better elucidate the consequences of mutations in *ANG* and/or *RNase 4* genes, in order to achieve early diagnosis and, possibly, successful therapies against ALS.

## 1. Introduction

### 1.1. Neurodegenerative Diseases, Including ALS, Are Associated to Aberrant Protein Aggregation

Several devastating neurodegenerative pathologies, such as Alzheimer’s, or Parkinson’s diseases (AD and PD, respectively), as well as Amyotrophic Lateral Sclerosis (ALS) [1] share uncontrolled protein aggregation events accompanied with the formation of amyloid deposits occurring either intracellularly or, less frequently, inside the cells of the affected individuals. These deposits consist of rigid, non-ramified cross-β-sheets fibrils formed by different proteins [2,3], which can be identified by Congo Red staining [4] or Thioflavin-T (ThT)-induced fluorescence [5]. Although amyloidogenic proteins do not share primary, secondary or tertiary structures, they all assume similar supramolecular organizations [2] that differ either in composition and intima structure of the short peptides forming the cross-β-sheet structures [6], or in the structure of the fibrils formed by the whole protein [7,8]. Indeed, many protein fibrils constantly reveal high structural polymorphism, as recently reported by studies exploiting cryo-EM flanking X-ray crystallography [9,10,11,12].

In addition, more than twenty years ago, the late Prof. Dobson and his co-workers assumed that any protein, given a high concentration and destabilizing environmental conditions, can form amyloid-like fibrils [13]. Importantly, the mature amyloid fibrillar plaques can be less toxic than their precursors, i.e., the soluble, pre-fibrillar aggregates [14], and the complex role of toxic oligomers has been recently reviewed [15]. This early effect principally occurs because the oligomers can inhibit fundamental cellular processes by interacting with cell membranes, thereby inducing oxidative stress, increasing Ca^2+^ concentration, and leading to apoptosis [16]. However, the clinical manifestation of one particular neurodegenerative disease, such as ALS, can be paralleled by similar co-morbidity(ies), making the scenario even more complicated [17,18,19].

Indeed, ALS is not easy to be efficacioulsy counteracted because it is a multifactor disease consequent to a progressive degeneration of motoneurons taking place either in the brain or in the spinal cord. Notably, no more than about 10% of ALS cases are currently listed as familial (fALS), while up to 90% are sporadic (sALS) [20]. Nevertheless, as illustrated by van Es et al. [21], many gene-related protein mutants are involved in the disease onset [20,22,23], and mutations of the gene encoding the natively dimeric Cu/Zn superoxide dismutase-1 (SOD1) are certainly present in about 20% of fALS patients [24]. In particular, in the European population, the most frequent ALS-related mutations affect, beyond *SOD1*, *C9orf72* intronic expansion, *TARDBP,* and *FUS* genes [25,26,27], as well as *ANG* [28]; however, ten years ago, more than 30 different genes were found to be associated with fALS [29].

The active role of SOD1 in ALS was discovered thirty years ago [30]. Upon undergoing aggregation [31], SOD1 affects the cytoplasm of motoneuronal cells, hence favoring the ALS income [24]. Since then, more than one hundred *SOD1* mutations have been associated with ALS, with A4S, H46R, and especially G93A being the most frequently studied, but also E22G, G37R or I113T [24,30,32,33,34,35]. Non-native SOD1 aggregation, or a simple impairing trimerization, has been also associated with ALS [36,37], while SOD1 amyloidogenic oligomers displayed polymorphism in the domain formed by residues of the 28–38 segment [38]. Finally, L84F-SOD1 has very recently been associated with fALS [39], and the Cys111 residue has revealed itself to be crucial in SOD1-related ALS cases [40].

In addition, the neuronal ubiquitinated cytoplasmic inclusions of the trans-active response DNA-binding 43 kDa protein (TDP-43) [41], encoded by the *TARDBP* gene, are linked to ALS. Indeed, since 2006 [41], many studies have revealed the key pathogenic effect of TDP-43 in ALS [42,43,44], as well as the role of mutations in facilitating the disease onset [45,46], such as the active role of the Q/N-rich C-terminal region [47], in line with the “poly-Q expansion” protein aggregation model proposed in 1999 by Sir Max Perutz [48]. Moreover, the abnormal phosphorylation of key-Ser residues [49], as well as other structural determinants, have been investigated for their ability to promote aggregation [50,51], while TDP-43 fibrils were also recently shown to be remarkably polymorphic [10,52,53]. Nevertheless, if on one hand, polymorphism makes the challenge more difficult, on the other hand, the use of natural, or “personalized” molecules or of nanobodies contrasting the formation of harmful amyloid deposits [54,55,56,57,58,59,60], or, even better, being able to disaggregate the pre-formed fibrils [60,61,62], could hopefully become a successful therapeutic strategy. However, and notably, one single protein can also be involved in different amyloidogenic pathologies, as it is for TDP-43, whose misfolding is often found, other than ALS, together with *C9orf72* intronic expansion in FrontoTemporal Dementia (FTD), which in turn drives to brain lobar degeneration [41,63].

Again, although not being the topic of this review, it must be recalled that neuroinflammation events consequent to protein aggregation are deleterious for cell life span and therefore cause the pathological manifestations of neurodegenerative diseases [64,65], like ALS [66]. Hence, the block of protein aberrant aggregation might consequently avoid, or reduce, these inflammatory consequences.

### 1.2. Physio-Pathological Roles of Human Angiogenin (ANG), Particularly in Neurological Contexts

**Figure 1:**
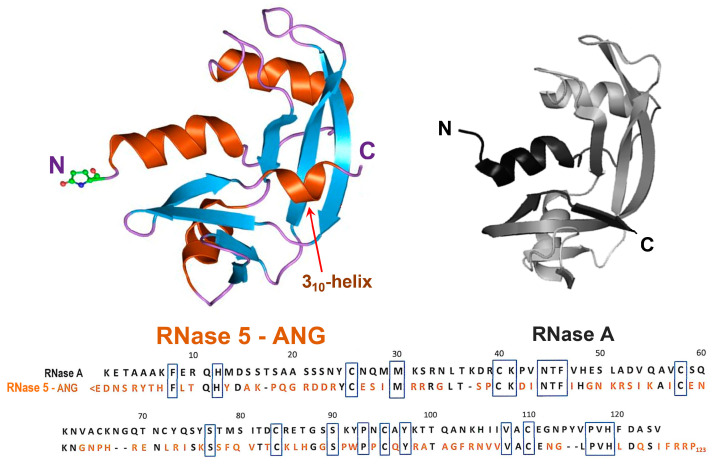
Upper panel: 3D structure of angiogenin (RNase 5—ANG, pdb code 1ANG) compared with the one of RNase A (pdb code 7RSA). In both structures, the N- and C-termini are indicated. In ANG, the N-terminal pyroglutamic residue is explicitly shown. Then, α-helices are shown in orange, as well as the 3_10_-helix tract indicated by a red arrow, while β-strands are shown in cyan and the flexible loops in purple. Lower panel: sequence alignment of the two RNase variants: where present, identity with RNase A (heading numbers refer to RNase A) is reported in black, while the different ANG residues are in orange. The black boxes indicate the residues conserved in all RNases of the super-family, comprising the H-K-H catalytic triad that in RNase A is numbered 12-41-119, while in ANG is 13-40-114.

In this complicated picture common to many neurodegenerative diseases, the role of the human secretory RNase 5 [67] in ALS must be highlighted beyond the proteins and factors mentioned so far. This particular basic ribonuclease is also called angiogenin [68] (ANG, Figure 1), and its connections with ALS represent the main topic of this review.

ANG can be compared to a two-faced medal: indeed, it plays physiological roles linked to its enzymatic activity, in this way stimulating tissues’ neo-vascularization and exerting a protective action for neuronal cells, or also other cells, against stress [69]. Again, ANG lowers the proliferation of human lymphocytes, hence becoming immunosuppressive [70], but can also exert antimicrobial activity [71]. Then, it displays antiviral activity by inhibiting HIV replication [72], although it apparently favors the replication of dengue virus [73].

On the other hand, however, ANG can induce pathological effects, like the growth of tumors’ mass thanks to its angiogenic tissues “feeding” [74,75]. Again, and notably, ANG is associated to PD, and especially to ALS onset upon undergoing Loss-of-Function (LoF) missense mutations [76]. Contrarily to other proteins, like the aforementioned SOD-1 and TDP-43, the role of ANG mutants in ALS seems to be ascribable to defects occurring in its enzymatic activity or cellular localization, and not to its possible aberrant aggregation. This seems true, although it was recently detected that ANG can be induced to dimerize [77], similarly to other RNases included in the same, so-called pancreatic-type (pt-RNase) super-family [78], whose proto-type is the well-known bovine pancreatic RNase A [79].

All the effects ascribable in ALS to ANG pathogenic mutants will be deeply analyzed in the following paragraphs. Finally, mutants of human RNase 4 [80], another angiogenic enzyme included in the pt-RNase superfamily [67,78], have also been found to be involved in the onset of ALS. A short paragraph in this review will list them.

## 2. Human Angiogenin (ANG), a Neuroprotective Ribonuclease, Acts as a Hormone Involved in Many Physiological Pathways

### 2.1. Main Features of ANG and Cell-Tissue Contexts in Which Its Ribonucleolytic Activity Is Exerted

RNase 5, or human angiogenin (ANG, Figure 1 left), belongs to the mentioned secretory pt-RNase superfamily, although displaying only 33% sequence identity with RNase A (Figure 1, right) [81]. Indeed, ANG actually shares, beyond its ribonucleolytic activity [82], four fundamental features with all pt-RNases known so far. In particular, (i) a similar V-shaped, or kidney-like folding (Figure 1) [83,84], accompanied by three out of four disulfide bonds characterizing RNase A-like enzymes; (ii) a His-Lys-His catalytic triad, composed of H13-K40-H114 [83], with the two His residues located in the N- and C-termini, respectively, and Lys in the enzyme core; (iii) the ability to cleave RNA substrates preferentially on the 3′side of pyrimidines like RNase A [82], following a two-step transphosphorylation/hydrolysis mechanism [79]; (iv) a CKxxNTF consensus sequence, comprising the catalytic Lys, as well as a PVH triplet at the C-terminus comprising the second catalytic His residue [85]. However, two other features characterize ANG: (i) its N-terminal pyroglutamic acid residue, shared only with RNase 4 [80] and amphibian onconase [86], and (ii) a 3_10_-helix tract involving the residues 117–121 that are located close to the ANG C-terminal end, which is a unique feature, absent in other pt-RNases [83].

Similarly to other secretory pt-RNases, the 5.4 kb *ANG* gene is located in the chromosome 14, precisely in the 14q11.2 locus [87], also encoding a leader 24AA residues-long sequence, or signal peptide preceding the mature 123 AA-residues-long ANG [88]. Hence, the numbering of ANG sequences sometimes considers these additional 24 residues as well [89]. The mRNA of ANG is present in many tissues, including the central nervous system (CNS) [87], while ANG was first characterized in 1985 as a 14.1 kDa monomer accompanied with ribonucleolytic activity [68,81] that is however lower than RNase A and other pt-RNases [82]. This reduced activity is principally ascribable to Glu116 and Phe120 [84], and, above all, to Gln117 residues that all partially obstruct the ANG active site cleft [83,90]. ANG was first characterized as a factor augmenting the tumor mass by inducing its vascularization and growth, since it was isolated and sequenced from human adenocarcinoma cell-conditioned media [68,81], or from hepatic tumors [75]. The low ribonucleolytic activity is however mandatory for ANG to exert its biological properties [91], like the activation of the growth factor in normal human tissues and plasma, or in the amniotic fluid [92,93]. Hence, it is a matter of fact to consider RNase 5 a hormone, and to call it angiogenin. Indeed, ANG promotes, at very low concentrations, and together with chemokines and factors like VEGF [94], the neo-formation of blood vessels [68,95]. It is also active in the ovarian follicle, as well as in the endometrium and placenta, with these tissues requiring extensive angiogenesis [96]. Hence, increasing levels of ANG are known to reflect the menstrual cycle phases, as well as the raised angiogenic request during pregnancy [97], and the development of the fetus blood vessels [98]. Again, ANG interacts with endothelial and smooth muscle cells, resulting in cell migration, invasion, proliferation and formation of tubular structures [99].

Last, but not least, the active role of ANG in the nervous system development and stability must be underlined: its effect starts very early, since ANG expression occurs in the axonal growth cones of mice embryos, then induces neurite growth [100]. ANG induces the activation of a Nrf2/antioxidant response in murine neurons [101], and is neuroprotective, in particular upon helping motoneuron survival [102,103].

### 2.2. Mechanistic Profiles and Molecular Targets of ANG Activity

**Figure 2:**
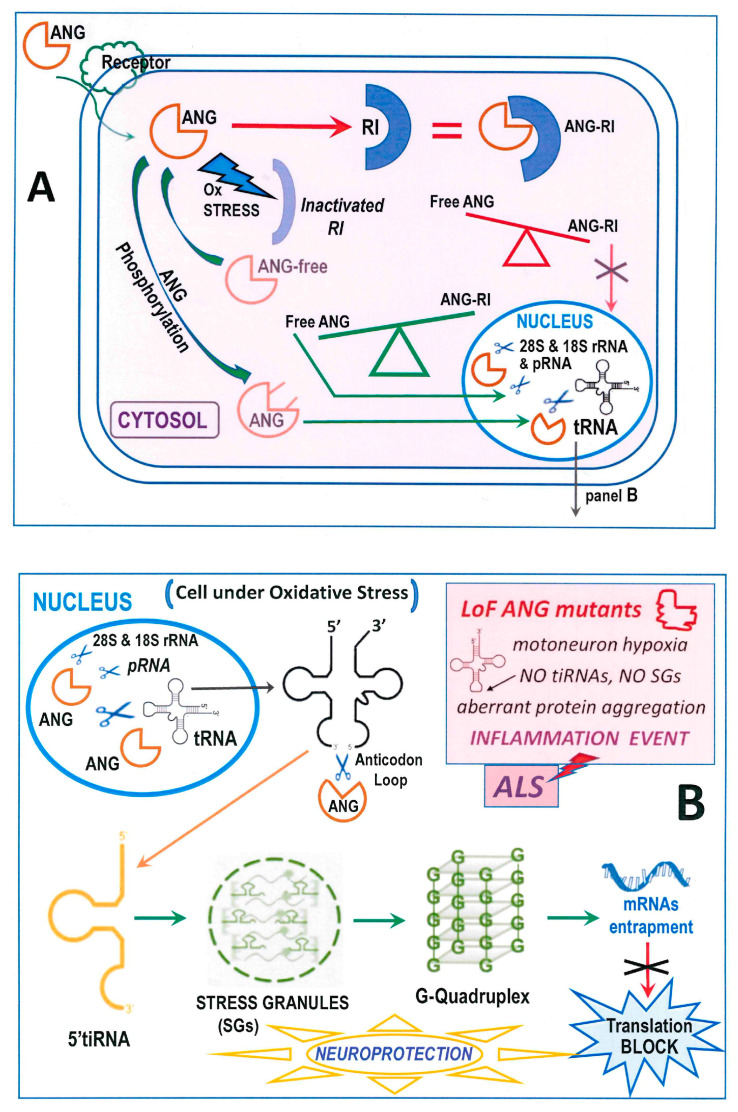
Schematic view of the cellular activity of RNase 5-ANG. (**A**): Cell entry and destiny of ANG under normal or, alternatively, under stress conditions that can in turn favor, or not, its entering the nucleus to attack RNA substrates; “RI” = Ribonuclease Inhibitor. (**B**) The neuroprotective scenario ascribed to the formation of SGs and G-quadruplexes following the production of the 5′tiRNA [123], half thanks to ANG enzymatic activity. The pathological neurodegeneration events ascribable to inflammation linked to ANG LoF mutations are summarized in the light-red squared inset.

From the report above, many biological activities are ascribed to ANG action [104]. These functions are exerted inside cells, in which ANG enters upon interacting with human cell-surface receptors, such as the poorly characterized 170 kDa protein factor [105], or the transmembrane heparan sulfate proteoglycan syndecan-4 that facilitates the ANG uptake into astroglia (Figure 2A) [103]. In particular, the portion comprised within the 60–68 residues forms the receptor-binding site that allows ANG to enter the cells [106]. Instead, in RNase A or RNase 1, the 60–68 peptide has a different sequence and is constrained by the Cys65-Cys72 disulfide [79]. Regardless, both RNase A and 1 can enter cells through endocytosis, but they are then sequestered by the cellular RNase Inhibitor (RI) [107], consequently forming a RI-RNase irreversible complex characterized by a K_D_ reaching femtomolar levels [108]. A similar, very strong complex is formed with RI also by ANG [108]. However, oxidative stress destabilizes RI [109], and consequently the cytoplasmic RI-ANG complex. This unbalances the ANG subcellular distribution, thereby affecting cell growth and survival (Figure 2A) [110]. Hence, amounts of ANG can be active also in the cytosol [110], and it has been recently reported that ANG exerts a cytoplasmic protective effect on skin keratinocytes suffering oxidative stress [111,112].

Alternatively, the RI-ANG complex formation is hindered by the phosphorylation of ANG Ser28, 37 and 87 residues, or of Thr36, operated by PKC or CDK [113], hence favoring ANG nuclear translocation (Figure 2A) [113]. Once entering the nucleus thanks to the ^31^RRRGL^35^ nuclear localization signal (NLS) containing three consecutive Arg residues, ANG accumulates into the nucleolus [114], where it can cleave pRNA, also defined NoRC-associated-RNA [113]. This substrate is a non-coding RNA that regulates the rDNA transcription of ribosomal rRNA [115] through the interaction with TIP5, a factor in turn critical for chromatin remodeling [116]. The cleavage of pRNA is an uncommon transcription pathway that impairs the remodeling cascade, in turn resulting in cell growth repression [113]. Moreover, since ANG can degrade both 28S and 18S rRNAs [82], its role overpasses rRNA transcription to become a rRNA processor as well (Figure 2A,B).

Importantly, tRNAs are the main targets of ANG as well [117] (Figure 2A,B). Indeed, ANG produces non-coding tiRNA derivatives at the anticodon loop that in the nervous system can induce functional and/or dysfunctional consequences [118]. Hence, although not all tRNAs are susceptible [119], the enzymatic activity of ANG affects crucial cellular pathways, like inhibiting protein translation [120]. ANG becomes active in the cytosol under stress conditions hindering its complexation with RI [109,111,112] and permitting the enzyme to reach and cleave the anticodon loop of tRNAs (Figure 2B). In this way, 3′ and a 5′tiRNA fragments are generated, causing the inhibition of protein translation [121]. The 5′tiRNA fragment leads instead to the assembly of stress granules (SGs) [122] that in turn induce the formation of G-quadruplexes. These structures exert a neuroprotective action [123] by trapping mRNAs and provoking translation arrest (Figure 2B). All these events do in turn contrast neuronal damages [102]. In more detail, the 5′tiANG halves deriving from ANG activity interact with cytochrome C, thereby protecting cells from apoptosis during osmotic stress [124]. However, it was reported that ANG forms higher amounts of tiRNAs under oxidative rather than osmotic stress because only oxidation dramatically decreases RI activity [125]. Interestingly, it was unveiled in neuronal SH-SY5Y cells that SGs induced by ANG activity can accumulate and consequently impair the activity of the A9D mutant of progranulin, that is in turn known to exert neurodegenerative effects (Figure 2B light-red inset) [126]. Conversely, the dysregulation of the composite stress response machinery involving ANG activity can drive neuronal cells death [127]. In particular, an excessive retention of neuronal mRNA can recruit high levels of TDP-43, thereby inducing its oligomerization and fibrillogenic aggregation [128]. The same authors hypothesized that, under stress, an unbalanced mRNA translation could trigger motoneuron damages at early ALS stages.

ANG can also cleave specific sequences of the TψC-loop of tRNAs to produce short harmful tRF-3 fragments [129]. This evidence is however debated, since other authors reported that ANG likely only acts at the tRNAs anticodon loop [130], whilst other RNases, like Dicer RNase III, are certainly known to cleave the TψC-loop [131]; hence, it can be envisaged that the environmental conditions might deflect ANG from its preferential cleavage at the anticodon loop. Indeed, tRFs fragments, comprising tRF-3, are shorter than tiRNAs and behave similarly to miRNAs, being crucial in many pathological contexts, like cancer [132], or viruses [133]. Furthermore, the possibility that tRFs might impair the neuroprotective activity of the longer 5′tiRNAs could complicate the scenario involving ANG, and for all the reasons reported ANG is considered an enzyme of substrate specificity, but with divergent functional abilities [104].

### 2.3. Neuroprotective Activity of ANG in the Context of ALS or Other Neurodegenerative Diseases

Native ANG can also act as a neuroprotective factor in pathologic contexts of CNS: indeed, native ANG is protective for neurons undergoing hypoxia, while its pathogenic mutants are not [134], and the wt-ANG concentration was found to be higher than its normal level in the serum of patients affected by ALS [135]. Moreover, wt-ANG promotes motoneurons’ survival either in vitro or in vivo in G93A-SOD1 ALS mice [136], while pathogenic ANG mutants do not [102]. Then, ANG undergoes selective uptake in astroglia where it can cleave RNA, contrarily to its K40I active-site mutant. This activity mediates the protection of neuronal cells in astroglia, hence highlighting the paracrine effect exerted by ANG [103]. Therefore, both ANG stability and enzymatic activity are mandatory for its protective profile, since it has been reported that poorly stable or low-activity ANG variants correlate with the early onset of ALS [137]. Surprisingly, however, the same study revealed that the loss of ANG stability and activity is proportional with a long ALS duration, while active ANG was shown to accelerate the effects of ALS and to shorten patients’ life-span [137].

The picture is therefore more complicated than hoped, and further investigations should clarify if the role of ANG in the disease can be beneficial or detrimental as a function of the pathology’s time course.

## 3. Impaired Levels of ANG Expression, and/or Missense Inactivating ANG Mutations Favor ALS Onset

### 3.1. General Aspects Depicting the Role of ANG in Neurodegenerative Pathologies

Several pathogenic mutants of many proteins are involved in the onset of ALS. The most important mutants of SOD-1 and TDP-43 have already been mentioned in a previous paragraph, with all of them undergoing extensive and uncontrolled amyloidosis directly involved in ALS-related cellular damage. In this paragraph, attention is devoted to the role of ANG in neurodegenerative pathogenesis, in particular on pathogenic mutants that, since about twenty years ago, were associated to ALS [138], as well as in some cases also with PD [76], but this falls out of the focus of this review.

We already described how native, wt-ANG can cleave the anticodon loop of tRNAs, thereby forming 5′tiRNA fragments being crucial to assemble neuroprotective SGs. However, since in humans more than 500 tRNA genes decode 61 tRNA codons, some of them can be precursors of non-coding short RNA fragments whose precise role needs further analysis [139]. Furthermore, many tRNAs undergo stabilizing post-translational modifications [140], such as an anticodon loop methylation, or ribose modifications that in turn impair the interaction with ANG. In this way, this peculiar RNase becomes unable to generate the mentioned 5′ti-RNA neuroprotective fragments [127,141]. Interestingly, an accurate review centered on the importance of small tRNA fragments in neurodegenerative disorders has been reported by Fagan et al. [118].

Pathogenesis, however, can arise from coupling a susceptible tRNA substrate with an inactive ANG variant. Indeed, although wt-ANG cleaves tRNAs under stress conditions inactivating RI [109], its main destiny is to be addressed into the nucleolus to trigger multistep pathways in turn inducing the neo-formation of vessels, either in healthy contexts or in cancer [68]. Therefore, the block of ANG activity to avoid cancer development would be likely accompanied with deleterious consequences, like the deletion of beneficial neuronal effects.

### 3.2. Pathogenic Mutations of ANG Related to Neurodegenerative Diseases, in Particular ALS

ANG exerts its RNase-related angiogenic activity in the nucleolus. Therefore, ANG mutations impairing its translocation into the nucleus, or that heavily limit or nullify its ribonucleolytic activity, surely hinder the neo-formation of blood vessels. Instead, if ANG were to suffer mutations impeding its interaction with RI, this might be potentially beneficial. However, as reported earlier, a higher or persistent RNase activity consequent to tRNA hypo-methylation for example [127] or to the inability of RI to block ANG also under normal conditions, could on one hand enhance the formation of SGs, but on the other hand affect the mRNA long-time retention that favors aberrant TDP-43 aggregation [128]. Consequently, the ribonucleolytic activity of ANG, and therefore its concentration and cellular localization, must be carefully balanced.

Coming back to ANG variants, nearly the totality of the pathogenic mutations induce LoF consequences. These mutations, located in the *ANG* gene, have often been associated with familial forms of neurodegenerative disorders, in particular ALS and PD [142,143]. The initial consideration that wt-ANG is highly concentrated in the serum of ALS patients is particularly interesting [135], but this abundance was first interpreted as a defense response exerted to protect neurons and motoneurons in an already compromised context ascribable to other factors [102,103].

**Figure 3:**
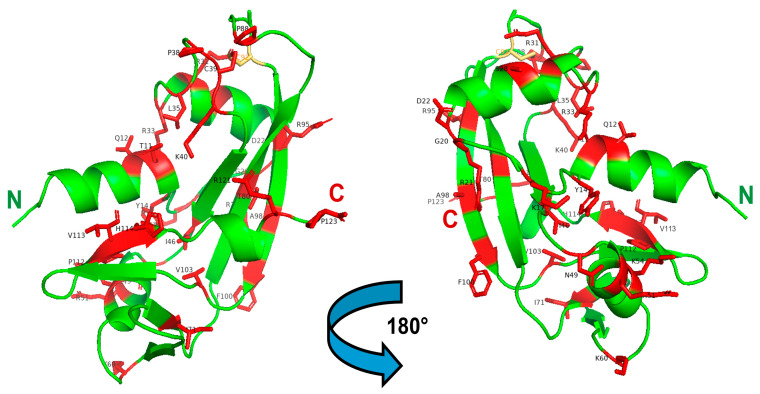
3D structure of ANG highlighting the registered ALS-related mutations. Left: front-oriented visualization; Right, 180°-turn back-oriented visualization. The 30 AA residues mutated in mature ANG, and listed in Table 1, are highlighted in red and labeled in black. The C92 residue that forms a disulfide with C39, in turn affected by the C > W pathogenic mutation, is shown in yellow. The N- and C-termini of the enzyme are labeled in green and red, respectively. The Figure was built with PyMol 2.4.1.

Instead, mutations affecting *ANG* were associated to ALS [28,144]. Additional studies listed increasing numbers of ALS-related *ANG* mutations, either in the coding region of the signal peptide or of the mature enzyme, as visible in Figure 3 and reported in Table 1. The first mutations associated to the onset of both fALS and sALS were Q12L, K17I/E, R31K, K40I, and I46V, all affecting mature ANG: these were found by Greenway et al. in Scottish, Irish, Irish/Scottish, and Irish/English patients, while C39W-ANG was registered in a wider European context [138]. The same authors hypothesized that *ANG* mutations might cause autosomal dominant ALS with low penetrance, while the I46V mutation was found also in some Italian ALS patients [145].

Crabtree et al. reported that all ANG mutants, except R31K, retain low RNase activity [146], since Q12L and especially K40I directly affect the catalytic site. Instead, this effect is indirectly exerted by C39W- and I46V-ANG, that are the less thermally stable variants. The K17I/E mutants were instead hypothesized to affect peripheral catalytic subsites, like in RNase A [79,147]. Then, although the R31K variant is enzymatically active, the however conservative R-to-K mutation resulted to be detrimental because the mutation affects the Arg triplet located in the NLS ^31^RRRGL^35^ peptide. In the same year, Wu et al. reported that P(-4)S, K17I, S28N and P112L *ANG* mutations, the first affecting the signal peptide, were found to be LoF, i.e., not angiogenic, in North American ALS patients [148]. Additionally, P112L-ANG was only somewhat enzymatically active, but unable to enter the nucleus, while the contrary was found to be true for K17I-ANG. Instead, S28N-ANG was again unable to enter the nucleus, although it retained a partial RNase activity. It also showed propensity to dimerize, and this latter feature was considered not detrimental for RNase activity [148]. Thereafter, other *ANG* mutations were associated with ALS in different populations, and synonymous mutations were detected as well, like 132C->T coding for G20G [149] or 497T->G for G86G [145]. In 2008, one Italian study informed of an ALS case accompanied with the M(-24)I mutation in the ANG signal peptide [150], while Gellera et al. involved a larger cohort of Italian ALS patients. Beyond G20G and M(-24)I, other *ANG* mutations encoding for F(-13)S and again P(-4)S, as well as the V113I and H114R mutations, were found in mature ANG [149]. H114R directly affects one catalytic residue, obviously inducing LoF consequences, while the V113I mutation modifies a residue inserted in the ^112^PVH^114^ tract that is conserved in all pt-RNases, although the corresponding variant was not functionally tested [85]. In the same year, some French patients presented, beyond the known I46V, a R121H mutation affecting the last residue of the ANG 3_10_-helix, hence likely altering the conformation of the enzyme catalytic cleft [151]. Again, three new, not further analyzed ALS-related *ANG* mutations, i.e., L35P, N49S and K60E, were discovered in Asian patients [152], while in 2009 the K54E-ANG mutant was discovered in a German patient [153]. McLaughlin et al. then analyzed how ANG levels and *ANG* genotypes dysregulation are crucial in Irish, Swedish and Polish ALS patients [154].

One year later, the R121C-ANG mutation, predicted to be harmful and reported as R145C because of the preceding 24AA signal peptide [88], was found in a *SOD1* G93D sALS Italian patient. This was the first case of the co-presence of mutations affecting both *SOD1* and *ANG* genes, thus inducing a speed-up of the disease progression [89]. The role of wt- or of ANG mutants in SOD1-related ALS contexts was therefore further analyzed [35,136]. Again, van Es et al. listed all the European and American nonsynonymous *ANG* mutations found in ALS, but also in PD patients: in particular, F(-13)L, G(-10)D, P(-4)Q, again K54E, T80S, and F100I [76], while *ANG* mutations coding for Y14H, D22G- and V103I-ANG were reported by two other ALS-related studies [155,156].

Then, Aparicio-Erriu and Prehn discussed the ANG paracrine activity occurring under stress conditions, like hypoxia or starvation, and on its impairment caused by *ANG* pathogenic mutations. They confirmed that secreted ANG accumulates in SGs (Figure 2B), thereby protecting motoneurons upon inducing an altered RNA metabolic profile in astrocytes [157]. The next year, new rare *ANG* mutations, i.e., G20S, P38R, R51H, P88H, R95L, A98V, and P123L emerged from other ALS patients [158]. Their possible role in affecting the conformational switch of the crucial catalytic H114 residue, as well the folding of the ^31^RRRGL^35^ nuclear localization ANG tract, was analyzed [159]. As a further consideration, it is worth mentioning that, while many cited ANG mutations are non-conservative, some of them involve Pro residues that can in turn affect proteins’ folding, as it occurs for example in RNase 1 [160].

Finally, three other rare *ANG* mutations, T11S, R21G, and I71V, respectively, emerged in Indian ALS patients [161,162,163], the second being LoF for either ribonucleolytic activity or nuclear translocation [162]. Again, the R33W mutation affecting the NLS ^31^RRRGL^35^ peptide was detected in the *ANG* gene of a Hungarian ALS patient [164].

All the ANG mutations mentioned in this paragraph are shown in Figure 3 and listed in Table 1.

**Table 1:** Nonsynonymous RNase 5 (ANG) mutations related to the onset of ALS.

N	Residue Mutated	Country (ies)	Functional Consequences	Reference(s)
1	M(-24)I	Italy	Not Determined	Conforti FL et al., 2008 [150]
2	F(-13)L	Germany/America	Not Determined	van Es MA et al., 2011 [76]
2 bis	F(-13)S	Italy	Not Determined	Gellera C et al., 2008 [149]
3	G(-10)D	Europe/America	Not Determined	van Es MA et al., 2011 [76]
4	P(-4)Q	Europe/America	Not Determined	van Es MA et al., 2011 [76]
4 bis	P(-4)S	North America	Not Determined	Wu et al., 2007 [148]/Gellera et al., 2008 [149]
5	T11S	India	Negative influence to catalytic residue H114	Padhi AK et al., 2019 [161]
6	Q12L	Scotland/Ireland	LoF *: enzymatic activity	Greenway MJ et al., 2006 [138]
7	Y14H	U.S.A.	Not reported	Brown JA et al., 2012 [155]/Zou ZY et al., 2012 [156]
8	K17I	IRE/Scotland/North America/GER/BEL/NED	LoF: enzymatic activity	Greenway et al., 2006 [138]/Wu et al., 2007 [148]
8 bis	K17E	Ireland/Sweden	Partial loss enzymatic activity	Greenway MJ et al., 2006 [138]
9	G20S	Not Reported	Probable enzyme activity loss	Padhi AK et al., 2013 [158]
10	R21G	India	LoF: partial enzymatic activity + nuclear translocation	Narain P et al., 2019 [162]
11	D22G	U.S.A.	LoF: enzymatic activity	Brown JA et al., 2012 [155]
12	S28N	North America	LoF: phosphorylation hinder/ Partial enzymatic activity LOSS	Wu D et al., 2007 [148]/Fasoli S et al., 2021 [77]
13	R31K	Ireland/England	LoF: NLS impairment	Greenway, 2006 [138]/Crabtree, 2007 [146]
14	R33W	Hungary	LoF: NLS impairment	Tripolski K et al., 2019 [164]
15	L35P	Asia	LoF: partial enzymatic activity	Takahashi Y et al., 2008 [152]
16	P38R	Not Reported	Probable enzymatic activity loss	Padhi AK et al., 2013 [158]
17	C39W	Europe	LoF: enzymatic activity and folding	[138] Greenway MJ et al., 2006
18	K40I	Ireland/England	LoF: catalytic site affected	Greenway MJ et al., 2006 [138]
19	I46V	Scotland/ITA/FRA/ GER/SWE	LoF: vdW contacts and enzymatic activity	Greenway, 2006 [138]/Corrado, 2007 [145]/ Paubel et al., 2008 [151]
20	N49S	Asia	LoF: enzymatic activity	Takahashi Y et al., 2008 [152]
21	R51H	Not reported	Not reported	Padhi AK et al., 2013 [158]
22	K54E	Germany/America	LoF: probably nuclear translocation	Fernandez-Santiago, 2009 [153]/van Es, 2011 [76]
23	K60E	Asia	No loss of enzymatic activity; not determined	Takahashi Y et al., 2008 [152]
24	I71V	India	Negative influence to catalytic residue H114	Padhi AK et al., 2021 [163]
25	T80S	Nederland/America	LoF: vdW contacts and enzymatic activity	van Es MA et al., 2011 [76]
26	P88H	Not reported	Probable enzymatic activity loss	Padhi AK et al., 2013 [158]
27	R95L	Not reported	Probable enzymatic activity loss	Padhi AK et al., 2013 [158]
28	A98V	Not reported	Not Determined	Padhi AK et al., 2013 [158]
29	F100I	Nederland/America	Partial enzymatic activity LOSS + flexibility	van Es et al., 2011 [76]/Zou ZY et al., 2012 [156]
30	V103I	China	Partial enzymatic activity LOSS + hydrophobicity	Zou ZY et al., 2012 [156]
31	P112L	North America	Partial enzymatic activity LOSS	Wu D et al., 2007 [148]
32	V113I	Italy	Steric hindrance for substrate bind	Gellera C et al., 2008 [149]
33	H114R	Italy	LoF: catalytic site affected	Gellera C et al., 2008 [149]
34	R121H	France	Gain of Function	Paubel A et al., 2008 [151]
34 bis	R121C	Italy (+G93D-SOD1)	Gain of Function	Luigetti M et al., 2011 [89]
35	P123L	Not reported	Not determined	Padhi AK et al., 2013 [158]

* LoF: Loss of Function.

### 3.3. Structure-Function Investigations Related to the Neuronal Pathogenic Effects of ANG Variants

In 2012 and 2017, the group led by K.R. Acharya carried out two pivotal studies reporting detailed 3D structure-function data affecting many of the mentioned ALS-related ANG mutants [165,166]; some ANG residues were already known to be crucial catalytic subsites stabilizing the interaction(s) of the enzyme with the substrate(s) [84].

Notably, many of these key residues exhibited conformational flexibility and dual conformations that are affected by LoF pathogenic mutations, thereby justifying the decreased RNase activity of ANG, or its inability to be translocated into the nucleus, or again to induce the formation of SGs. Instead, the R121H/C variants exhibited increased RNase activity [165], as was confirmed later in our studies [77]. Interestingly, the increased RNase activity of the R121H/C-ANG mutants is likely ascribable to the decreased hindrance caused by the modified side-chain. This affects the interaction of the 121 AA-residue with Ser118 [165,166], which is in turn part of the B_2_ catalytic subsite [83]. The apparently paradoxical pathogenic effect due to an ANG-augmented catalytic activity was tentatively associated with the increased effect induced by R121H-ANG in neurite cultures with respect to the wt [165]. Alternatively, the already cited studies performed by Colombrita et al., or by Aluri et al. indicate that both the persistence and the increased RNase activity in ALS patients could switch the effect of ANG from protective to detrimental [128,137].

Thiyagarajan et al. also reported that the Q12L mutation affects crucial interactions with L10 and T44 [165], that are in turn part of the ANG P_1_ catalytic subsite involved in the phosphodiester bond cleavage [83], while the more frequent I46V mutation deletes many van der Waals contacts involving some residues of the catalytic site located in the ANG amino-terminal α-helix [165]. A similar loss of vdW contacts, in this case involving the T44 subsite, is caused by the T80S mutation too [166]. Instead, the additional methyl group of the V113I mutant sterically hinders the substrate binding, accounting for the relative decreased RNase activity. Moreover, the P112L mutation, beyond its closeness to the B_2_ subsite interacting with a purine base located on the opposite site of the scissile bond [83], turned out to also affect the interaction with NLS, therefore impairing ANG nuclear translocation similarly to K17I-ANG [165]. This latter aspect was also associated to the effect exerted by the S28N mutation [165], considering that the corresponding variant loses the interaction with Arg32, which is part of the NLS ^31^RRRGL^35^ sequence [114]. Instead, both F110I- and V103I-ANG displayed altered flexibility, or stabilizing interactions involving key enzyme regions, respectively [166]. Finally, the mutations of catalytic site residues, i.e., K40I, and H114R, or directly affecting the NLS sequence, like R31K, or again the stability of the enzyme, like K17I/E and C39W, were easily associated with LoF consequences [165].

Importantly, the already cited study carried out by Hoang and Raines revealed that the pathogenicity of S28N-ANG mutant can be ascribed to its scarcely phosphorylated level in the cell, thereby resulting in the cytosolic RI-ANG complexation, that in turn hinders the enzyme nuclear translocation [113]. Afterwards, the analysis of C39W and R121C *ANG* mutations inducing the hindering of SGs formation that in turn blocks the neuroprotective G-quadruplexes deriving from 5′-tiRNA^Ala^ fragments [123], suggested that the more active R121C-ANG may induce a partial entrapment of the fragments as a duplex form, thereby retarding the G-quadruplex formation [167]. However, additional investigations are necessary to confirm this latter hypothesis.

## 4. Possible Physio-Pathological Effects of ANG Self-Association

### 4.1. Oligomerization of Secretory pt-RNases and Their Potential Amyloidogenic Role

Some secretory basic endoribonucleases, in particular the most studied bovine pancreatic monomeric RNase A [168], can be induced to form dimers [169,170], trimers [171,172], and larger oligomers [171,172,173] through the intertwining, or the so-called 3D domain swapping (3D-DS) [174] of the N- and/or C-termini [169,170] of two or more protomers [169,170,172,173]. At least two conformers per oligomer are formed [169,170,172,173,174], all of them retaining or augmenting the ribonucleolytic activity of native RNase A [175], since their active site is reconstituted by the diverse subunits undergoing 3D-DS [169,170,172,173]. Then, the RNase A oligomers often display peculiar biological actions, in particular an anti-tumor activity exerted both in vitro and in vivo [175,176] principally because they can sterically evade RI [177].

These oligomers share some structural features [169,170] with amyloidogenic proteins: in fact, RNase A mutants displaying a Gly_2–6_ peptide elongation between the N-term-swapped domain and the protein core, or a Gln_10_ extension before the C-terminus, spontaneously produce amyloid-like fibrils through 3D-DS [178,179], similarly to proteins undergoing fibrillation through the same mechanism [180].

Although wt-RNase A is not amyloidogenic per se, some short peptides of it spontaneously form “steric-zipper cross-β-spine” polymorphic structures [6,181]. However, and notably, fibrils have also recently been detected from wt-RNase A when the enzyme overpasses the supersaturation barrier [182], or when it acts in macromolecular crowding environments [183], that can in turn also affect the extent of RNase A-controlled oligomerization [184]. Again, the RNase A N-term-swapped oligomers, whilst neither the C-term-swapped ones, nor the monomer, can promote the slow formation of “super-aggregates” [185] that resemble the protofibrils detected in crowding contexts [183].

Importantly, 3D-DS oligomerization also involves other pt-RNases. Indeed, human pancreatic RNase 1 can reach an even higher self-association extent than bovine RNase A [186], while some mutants constitutively form N-term-swapped dimers [187,188], in some cases potentially prone to undergo fibrillization [188]. These RNase 1 mutant N-swapped dimers of RNase 1 are similar, but not identical to bovine seminal (BS)-RNase, i.e., the unique natively N-swapped and antitumor dimer of the pt-RNase superfamily [189,190]. Then, the eosinophil cationic protein (ECP), i.e., RNase 3 [67], a variant that can exert neurotoxicity [191], displays a N-terminal region prone to undergo amyloidosis [192]. Again, another constitutively monomeric but antitumor RNase, i.e., the already cited amphibian onconase [86], folds similarly to RNase A despite having only 30% sequence identity [193], and is also able to form cytotoxic dimers through the swapping of its N-termini [194,195].

Importantly for the topic of this review, also ANG, or RNase 5, was recently shown to dimerize through the swapping of its N-termini [77]. In addition, a couple of ANG mutants found in patients affected by ALS or PD displayed to dimerize at a slightly higher extent than the wt [77]. Hence, a direct role of ANG pathogenic mutants in the formation of harmful neurodegenerative aggregates and fibrils could be at first envisaged. However, in the previous paragraphs, the effects of ANG in ALS onset and development were depicted to be multifaced and complicated.

### 4.2. Effects of ANG Oligomerization on Its Enzymatic Properties and Pathological Consequences like ALS

Wt-ANG was analyzed for its tendency to oligomerize similarly to RNase A [175] in parallel with two ALS-related ANG mutants, in particular the less enzymatically active S28N-ANG variant, that had been previously considered prone to dimerize [148], and the more enzymatically active R121C-ANG: either wt or the two ANG variants were shown to dimerize through the swapping of their N-termini, as confirmed by different experimental approaches [77], while, notably, both mutants dimerized at a higher extent than the wt. Again, all dimers maintained an enzymatic activity similar to the one of their relative monomers. Instead, neither the ANG mutants nor the wt underwent massive aggregation or fibrillation [77].

Hence, although many other mutants could and should be tested, this result suggests that ANG mutants do not produce harmful aggregates, differently from the previously mentioned SOD1 and TDP-43 pathogenic variants. However, since the conservation of the structural and functional properties of ANG can be dramatically modified by even slight modifications related to particular mutations [165], the different propensity to form dimers might somehow modify the functional properties of the enzyme in vivo, thereby affecting either ANG nuclear translocation or enzymatic efficacy. Incidentally, it has been recently reported that ANG can form a non-swapped dimer upon associating with a ds-RNA duplex, although this event has been registered only in vitro [196]. Nevertheless, the structural arrangements consequent to the interaction of ANG with RNAs, and the possible formation of dimeric or oligomeric derivatives could somehow affect the properties of the native enzyme. To this end, I recall here that a nonnative SOD1 trimer involved in ALS displayed to be toxic to motoneurons, contrarily to the SOD1 native dimer [37].

Finally, a structural model depicting the cytoplasmic interaction of ANG with the proliferating cell nuclear antigen (PCNA) [197] indicated that three ANG subunits separately interact with each one of the subunits of a cyclic PCNA trimeric assemble [198]. The authors suggest that this particular supramolecular assembly might further depict the biological roles of both ANG and PCNA in the cytoplasm. However, and finally, all these spot-like findings involving ANG oligomerization, or supramolecular organization, require further investigation, especially in the context of ALS.

## 5. RNase 4 Mutants Involved in ALS Onset and Development

Although this review is principally centered on the ANG-ALS connection, some other mutants of RNase 4, another member of the pt-RNase superfamily, were found to favor the ALS onset [199]. Being only 119 AA-residues long [80], RNase 4 is the smallest member of the eight “canonical” human pt-RNase groups numbered 1–8 [67]. It displays about 40% identity sequence either with both RNase A and RNase 1, or with ANG, as well as a conserved H12-K40-H116 catalytic triad [80], and it is characterized by all the features characterizing pt-RNases, like, above all, a similar V-shaped 3D structure [200].

RNase 4 is selective for 3′-uridines [200] and displays a promoter peptide, as well as a N-terminal residue a pyroglutamic acid, both features the same as ANG, with which it is also co-expressed [201].

Again, RNase 4 is protective against urinary inflammations [202]; however, conversely, it was found to favor prostate cancer cell proliferation [203].

Comparably to ANG, RNase 4 is an important angiogenic [204] and neuroprotective factor [199], Neuroprotectivity is surprisingly exerted also by its K40A mutant, considering that it retains only a slight ribonucleolytic activity [199]. Instead, the T(-13)S, R10W, E48D, V75I, and A98V mutations affecting *RNase 4* have been surely associated with sALS [199]. The molecular dynamics of these mutants have been subsequently analyzed to better elucidate their structural features and their role in favoring the onset of ALS [205].

The *RNase 4* gene is also affected by other LoF ALS-related mutations: in particular, D2E, N26K, T79A, and G119S [161]. Again, M29I, R31T, R32W, H72P, and R95W are LoF mutations, while the other three impair the interaction of the substrate(s) with the catalytic H116 residue [206]. No other studies further analyzed the RNase 4 role in ALS, but the ones performed often reported structure-function relationships similar to ANG-ALS.

## 6. Conclusions and Possible Translation to Therapeutic Approaches

The present review illustrates how the biochemical properties of ANG are at the same time peculiar and difficult to finely regulate as well. The hormonal active role of RNase 5 ANG in the neo-formation of vessels overpasses its ribonucleolytic activity, that must be in turn precisely tuned to permit the enzyme to exert its physiological activities.

Indeed, LoF mutations affecting *ANG* (and also *RNase 4*) listed here can be crucial in triggering neurodegeneration. In particular motoneuron hypoxia and death, that in turn pave the way to the ALS onset, together, or in parallel with other factors.

We additionally illustrated, however, that an excessive ANG ribonucleolytic activity can also become detrimental for patients’ survival, especially when they are affected by ALS forms that are associated with SOD1 or TDP-43 pathogenic mutants [137]. Nevertheless, studies analyzing this latter aspect are still rare and further investigations are required to better understand how the neuroprotective effects of ANG can be correctly balanced to avoid its undesired conversion to toxic, pathological consequences.

All these arguments suggest that successful strategies devoted to therapeutically counteract the harmful ANG-ALS effects are not easy to be planned. However, since ANG is certainly pathogenic upon suffering mutations, like SOD1 and TDP-43, a gene-therapy approach could be promising. Certainly, a wide genetic screening could reach individuals prone to be affected by the disease, although it would be very difficult and expensive to plan a large-scale analysis. Hence, a narrowed approach involving people affected by other ALS-inducing factors could better focus a successful targeted strategy.

However, and finally, additional studies are required to better comprehend the positive or negative contribution of ANG in ALS and to state its correct and balanced sub-cellular distribution permitting to avoid a switch from physiological to pathological effects.
